# A Recombinant Human Pluripotent Stem Cell Line Stably Expressing Halide-Sensitive YFP-I152L for GABA_A_R and GlyR-Targeted High-Throughput Drug Screening and Toxicity Testing

**DOI:** 10.3389/fnmol.2016.00051

**Published:** 2016-06-28

**Authors:** Katharina Kuenzel, Oliver Friedrich, Daniel F. Gilbert

**Affiliations:** ^1^Department of Chemical and Biological Engineering, Institute of Medical Biotechnology, Friedrich-Alexander-Universität Erlangen-NürnbergErlangen, Germany; ^2^Erlangen Graduate School in Advanced Optical Technologies, Friedrich-Alexander-Universität Erlangen-NürnbergErlangen, Germany

**Keywords:** YFP-I152L, glycine receptor chloride channel (GlyR), gamma-aminobutyric acid receptor type-A chloride channel (GABA_A_R), human pluripotent embryonal teratocarcinoma stem cells, NT2 cells, NT2-N cells

## Abstract

GABA_A_Rs and GlyRs are considered attractive drug targets for therapeutic intervention and are also increasingly recognized in the context of *in vitro* neurotoxicity (NT) and developmental neurotoxicity (DNT) testing. However, systematic human-specific GABA_A_R and GlyR-targeted drug screening and toxicity testing is hampered due to lack of appropriate *in vitro* models that express native GABA_A_Rs and GlyRs. We have established a human pluripotent stem cell line (NT2) stably expressing YFP-I152L, a halide-sensitive variant of yellow fluorescent protein (YFP), allowing for fluorescence-based functional analysis of chloride channels. Upon stimulation with retinoic acid, NT2 cells undergo neuronal differentiation and allow pharmacological and toxicological evaluation of native GABA_A_Rs and GlyRs at different stages of brain maturation. We applied the cell line in concentration-response experiments with the neurotransmitters GABA and glycine as well as with the drugs strychnine, picrotoxin, fipronil, lindane, bicuculline, and zinc and demonstrate that the established *in vitro* model is applicable to GABA_A_R and GlyR-targeted pharmacological and toxicological profiling. We quantified the proportion of GABA_A_R and GlyR-sensitive cells, respectively, and identified percentages of approximately 20% each within the overall populations, rendering the cells a suitable model for systematic *in vitro* GABA_A_R and GlyR-targeted screening in the context of drug development and NT/DNT testing.

## Introduction

GABA type-A receptors (GABA_A_R) and strychnine-sensitive glycine receptors (GlyR) are ligand-gated chloride ion channels that mediate inhibitory neurotransmission in the central nervous system (CNS). In adult neurons, GABA_A_R and GlyR ion channels conduct an inhibitory anion current, mainly carried by chloride (Cl^−^) upon activation by γ-aminobutyric acid (GABA) and the amino acid glycine, respectively. In embryonic neurons however, due to a higher intracellular chloride concentration compared to adult neurons, receptor activation causes an outward directed, depolarizing and excitatory Cl^−^ flux (Webb and Lynch, 2007). GABA_A_Rs and GlyRs are both members of the pentameric ligand-gated ion channel (pLGIC) family and require five subunits to form a single functional oligomer. For GABA_A_Rs there are 19 genes known (α1–6, β1–3, γ1–3, δ, ε, θ, π, and ρ1–3) exhibiting a broad range of heterogeneity and many hundreds of theoretically possible subunit combinations (Olsen and Sieghart, 2009). For GlyRs there are four genes known (α1–3, β) in humans, exhibiting far less diversity compared to GABA_A_Rs (Lynch, 2009). Each GABA_A_R or GlyR isoform as well as each subunit combination has a unique physiological and pharmacological profile. The subunit combination can change during development, in a tissue-specific manner or as a consequence of pathophysiological events (Lynch, 2004; Webb and Lynch, 2007; Esmaeili and Zaker, 2011; Rudolph and Möhler, 2014; Deidda et al., 2015). Genetic or molecular perturbation of the channels' function has been associated with severe neurological disorders including neuropathic pain (Lian et al., 2012; Xiong et al., 2012; Chen et al., 2014), chronic pain sensitization (Harvey et al., 2004; Zeilhofer, 2005; Lynch and Callister, 2006), hyperekplexia (Chung et al., 2010; Bode and Lynch, 2014), epilepsy (Meier et al., 2005; Eichler et al., 2008, 2009; Macdonald et al., 2010), fragile X mental retardation syndrome (D'Hulst et al., 2006), learning and memory deficits (Deidda et al., 2015) as well as neurodegeneration (Kang et al., 2015). In addition, GABA_A_Rs and GlyRs are increasingly acknowledged in the context of immunomodulation (Stoffels et al., 2011; Gunn et al., 2015), amyotrophic lateral sclerosis (Martin and Chang, 2012) and cancer (Neumann et al., 2004; Cuddapah and Sontheimer, 2011). Therefore, GABA_A_Rs and GlyRs, including individual isoforms as well as the various subunit combinations in their native neuronal environment are increasingly considered highly attractive drug targets for therapeutic intervention (Alexander et al., 2015). Due to their fundamental role in inhibitory neurotransmission GABA_A_Rs and GlyRs are increasingly recognized in the context of neurotoxicity (Suñol et al., 1989; Hall and Hall, 1999; Narahashi, 2002; Vale et al., 2003; Mohamed et al., 2004; Islam and Lynch, 2012) and *in vitro* neurotoxicity testing (NT) (Talwar et al., 2013; Tukker et al., 2016).

Although GABA_A_Rs and GlyRs play fundamental roles during brain development (Avila et al., 2013, 2014), these receptors have only sparsely been associated with developmental neurotoxicity and developmental neurotoxicity (DNT) testing. This is even more surprising as the incidence of neurological diseases including learning and developmental disorders has increased in recent years (May, 2000; Colborn, 2004; Rauh et al., 2006; Herbert, 2010). At the same time, the number and volume of worldwide registered and traded chemical substances has also increased. There is no doubt that developing brain is particularly vulnerable to damage by chemicals (Rice and Barone, 2000) and evaluation of chemicals for developmental neurotoxicity is critical to human health (Grandjean and Landrigan, 2006, 2014). However, only a very small number of chemicals has been tested for developmental toxicity in recent years (Middaugh et al., 2003; Makris et al., 2009), presumably because the current guidelines for DNT testing exclusively involve animal experiments (OECD, 1997, 2007) that are of poor reproducibility and predictive quality, low in throughput, prohibitively expensive and limited with regard to mechanistic insights into the toxicant's mode of action (Smirnova et al., 2014).

DNT testing is conducted for identification of chemical-induced adverse changes in the structure and function of the developing central nervous system. At present, NT and DNT testing is only officially acknowledged by regulatory authorities when done with standardized *in vivo* animal test methods and when conducted according to guidelines provided by the *Organization for Economic Cooperation and Development* (OECD). For example, NT testing involves daily oral dosing of rats for acute, sub chronic or chronic assessments for 28 days, 90 days, 1 year or longer (OECD, 1997). Primary observations include behavioral assessments and evaluation of nervous system histopathology. DNT testing evaluates *in utero* and early postnatal effects by daily dosing of at least 60 pregnant rats from implantation through lactation. Offspring are evaluated for neurologic and behavioral abnormalities and brain weights and neuropathology are assessed at different times through adulthood (OECD, 2007). The type of exposure (single or repeated dose) and the outcome (lethal or nonlethal; immediate or delayed effects) will result in different classifications for substances under the Globally Harmonized System (GHS).

Since there are various methods available for toxicological profiling of GABA_A_Rs and GlyRs (Gilbert et al., 2009a,b,d; Talwar et al., 2013) these receptors can serve as valuable molecular targets for *in vitro* developmental neurotoxicity testing (DNT) and provide mechanistic insights into the neurotoxicants or developmental neurotoxicants mode of action.

However, systematic screening for potentiating or inhibiting modulators of GABA_A_Rs and GlyRs in the context of drug development and NT/DNT testing is hampered due to lack of appropriate *in vitro* models. Recombinant expression systems using e.g., human embryonal kidney-derived (HEK293) cells allow systematic large scale screening for GABA_A_R and GlyR modulators in high throughput format (Kruger et al., 2005; Gilbert et al., 2009a,b,d; Talwar et al., 2013; Walzik et al., 2015). Despite recombinant models being successful in the identification of GlyR chloride channel modulators (Balansa et al., 2010, 2013a,b), these systems lack of fundamental neuronal genetic programs and cell intrinsic regulators influencing the functional properties of mature neurons *in vivo* and are restricted to physiological, pharmacological and toxicological analysis of individual GABA_A_Rs and GlyRs isoforms in isolation. Modulators identified or investigated using recombinant expression systems have been reported to yield contradictive results comparing recombinant systems and native neurons. For example, NV-31 an analog of bilobalide, a major bioactive component of Ginkgo biloba herbal extracts, has been reported to inhibit recombinant GlyRs but to potentiate native hippocampal neuron GlyRs (Lynch and Chen, 2008). Hence, screening data generated using recombinant expression system may be only partially relevant to GABA_A_Rs and GlyRs expressed *in vivo* and always require time and resource intensive retesting using secondary and individual approaches. Terminally differentiated neuronal cells of human origin, e.g., primary cells from biopsy samples are rarely available, enable only a limited number of experiments and are typically derived from pathogenic tissue, rendering these cells unsuitable to systematic large-scale screening for modulators of GABA_A_Rs and GlyRs. Neuronal cells of animal origin such as mouse or rat are widely used for studying mammalian inhibitory neurotransmission in general and the physiological properties of GABA_A_Rs and GlyRs in particular but are not optimal for identification of human-specific therapeutic leads or pharmacological probes as well as for GABA_A_R and GlyR mediated neurotoxicity or developmental neurotoxicity as the physiology of animals may strongly differ from human physiology. Stem cells including induced pluripotent stem cell (iPSC) and pluripotent embryonal carcinoma cells provide an enormous potential for both GABA_A_R- and GlyR-targeted drug development and NT/DNT testing as they allow standardized high-throughput *in vitro* screening of a large number of chemicals in maturing and adult human neurons, that is time, cost and resource-effective.

Human pluripotent NTERA-2 (NT2 or TERA2.cl.SP12) stem cells are increasingly considered as a suitable model for *in vitro* NT and DNT studies (Couillard-Despres et al., 2008; Hill et al., 2008; Laurenza et al., 2013; Pallocca et al., 2013; Stern et al., 2014). Upon exposure to retinoic acid, the cells undergo neuronal differentiation, i.e., mimic the process of differentiation in the developing brain, and are potentially suitable to NT/DNT testing at different developmental stages ranging from non-differentiated stem cells, committed neural progenitors to differentiated neuronal, so called NT2-N cells, and glial cells (Lee and Andrews, 1986; Pleasure et al., 1992; Sandhu et al., 2002; Stewart et al., 2003; Ozdener, 2007; Coyne et al., 2011). Electrophysiological studies and extracellular recordings with NT2-N cells have demonstrated voltage-activated calcium, TTX-sensitive sodium and potassium currents, spontaneous synaptic currents as well as glutamate, N-methyl-D-aspartate (NMDA), GABA and strychnine-sensitive glycine-induced currents (Pleasure et al., 1992; Munir et al., 1996; Neelands et al., 1998; Gao et al., 2004; Coyne et al., 2011; Laurenza et al., 2013) demonstrating that these cells exhibit properties similar to those described in native human neurons thus, making them an excellent experimental model for both GABA_A_R- and GlyR-targeted drug development and NT/DNT testing. Also, a variety of neuronal markers has been reported to be expressed in differentiated NT2-N cells, including β-tubulin type III, MAP-2 and synapsin I (Pleasure et al., 1992; Stewart et al., 2003; Hsu et al., 2014; Saporta et al., 2014; Stern et al., 2014).

To address the limitations of conventional *in vitro* models for GABA_A_R- and GlyR-targeted drug, NT/DNT screening as well as of animal-based *in vivo* NT/DNT testing approaches described above, we aimed to establish a cell line stably expressing YFP-I152L under the control of the human ubiquitin promoter C. The promoter has been reported to drive selective protein expression in principal neurons in the mammalian brain (Wilhelm et al., 2011). NT2 cells have previously been reported to provide a suitable system for expressing exogenous proteins in terminally differentiated neurons (Pleasure et al., 1992).

YFP-I152L, an engineered variant of yellow fluorescent protein (YFP) with greatly enhanced anion sensitivity, is quenched by small anions and is thus suited to reporting anionic influx into cells (Galietta et al., 2001). The fluorescent protein has been successfully applied for structure-function analysis and compound screening with many different chloride channel types (Kruger et al., 2005; Gilbert et al., 2009a,b,d; Balansa et al., 2010, 2013a,b; Chung et al., 2010; Gebhardt et al., 2010; Talwar et al., 2013; Walzik et al., 2015).

We further aimed to apply the cell line in concentration-response experiments with GABA and glycine as well as with a selection of chemicals with known toxicity profiles on GABA_A_Rs and GlyRs and to compare GABA and glycine EC_50_ and drug IC_50_ values with published electrophysiological data and data from fluorescence based functional imaging.

To evaluate the suitability of the *in vitro* model to systematic large-scale functional screening in the context of GABA_A_R- and GlyR-targeted drug development and NT/DNT testing, we intended to quantify the proportion of GABA_A_R- and GlyR-positive cells.

Our *in vitro* model and methodological approach will be applicable within a framework of various individual strategies assessing NT/DNT at different stages during neuronal differentiation as well as to systematic large-scale *in vitro* neurophysiological, -pharmacological and -toxicological screening with GABA_A_Rs and GlyRs that is time, cost and resource-effective. Furthermore, in the context of *in vitro*-based experimental and analytical approaches for NT/DNT prediction, our methodology can contribute to reduce or even replace animal experiments and to further promote the concept of the 3Rs in biomedicine (Russell and Burch, 1959).

## Results

**Figure 1:**
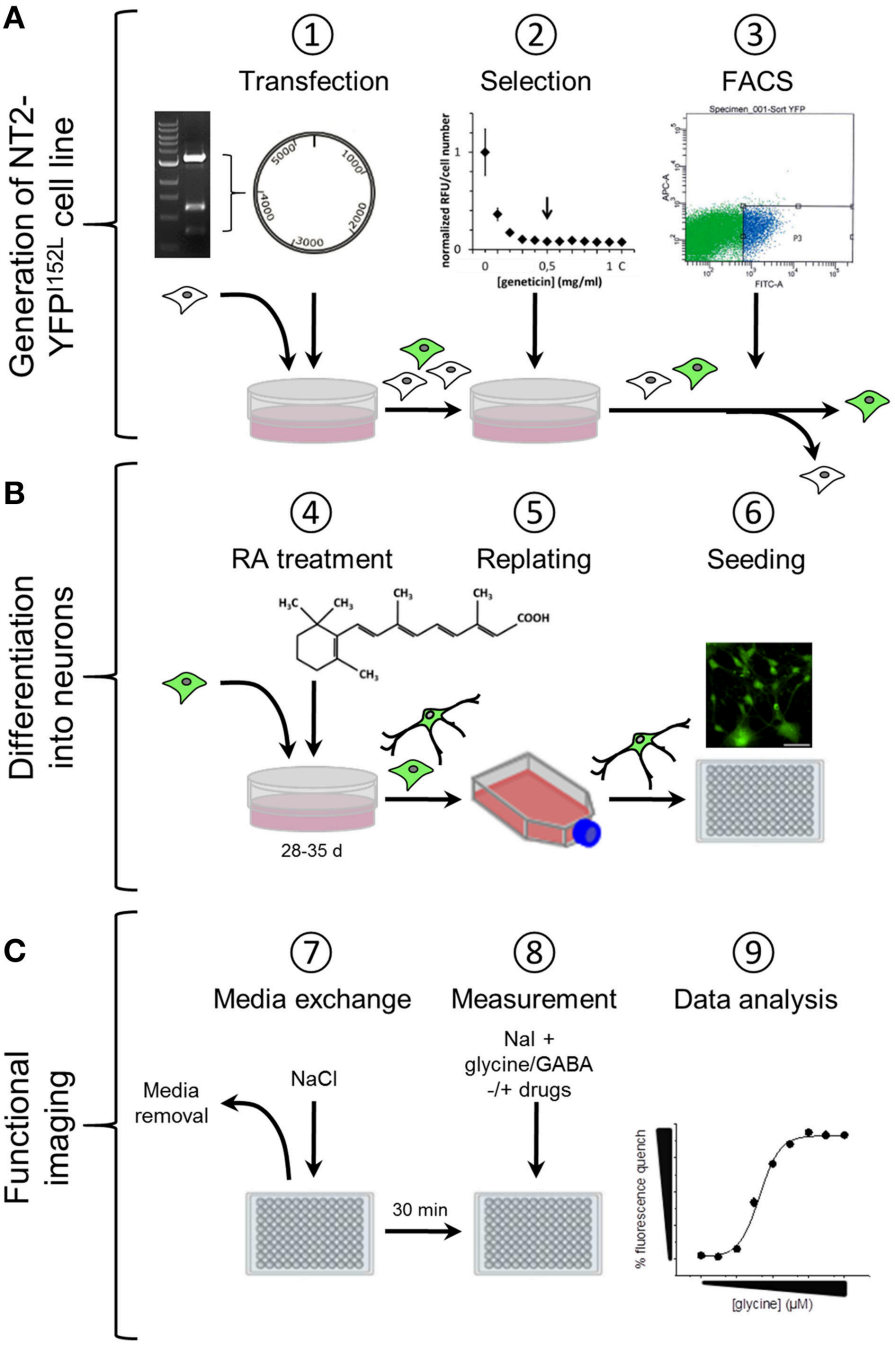
**Workflow of cell line generation, stem cell differentiation and functional imaging. (A)** Generation of NT2-YFPI52L cell line. NT2 stem cells were seeded into 60 mm culture dishes and transfected with ubiquitin-YFP-I152L-encoding vector using the calcium phosphate precipitation method. YFP-I152L-positive cells were selected over 5 weeks using the antibiotic geneticin. Subsequently, NT2-YFP-I152L cells were enriched by flow cytometry (FACS). **(B)** Neuronal differentiation of human pluripotent NT2 stem cells and preparation for functional imaging. Cells were differentiated based on 5-week retinoic acid (RA) treatment and NT2-N cells were enriched by a double-replating strategy. As a preparatory step for imaging of GABA_A_R and GlyR-function, cells were plated into 96-well plates. **(C)** Preparation of imaging experiments. Approximately 30 min prior to imaging experiments, the culture media was removed and replaced by control solution supplemented with pharmacological drugs. Functional imaging was conducted as shown in Figure 2.

**Figure 2:**
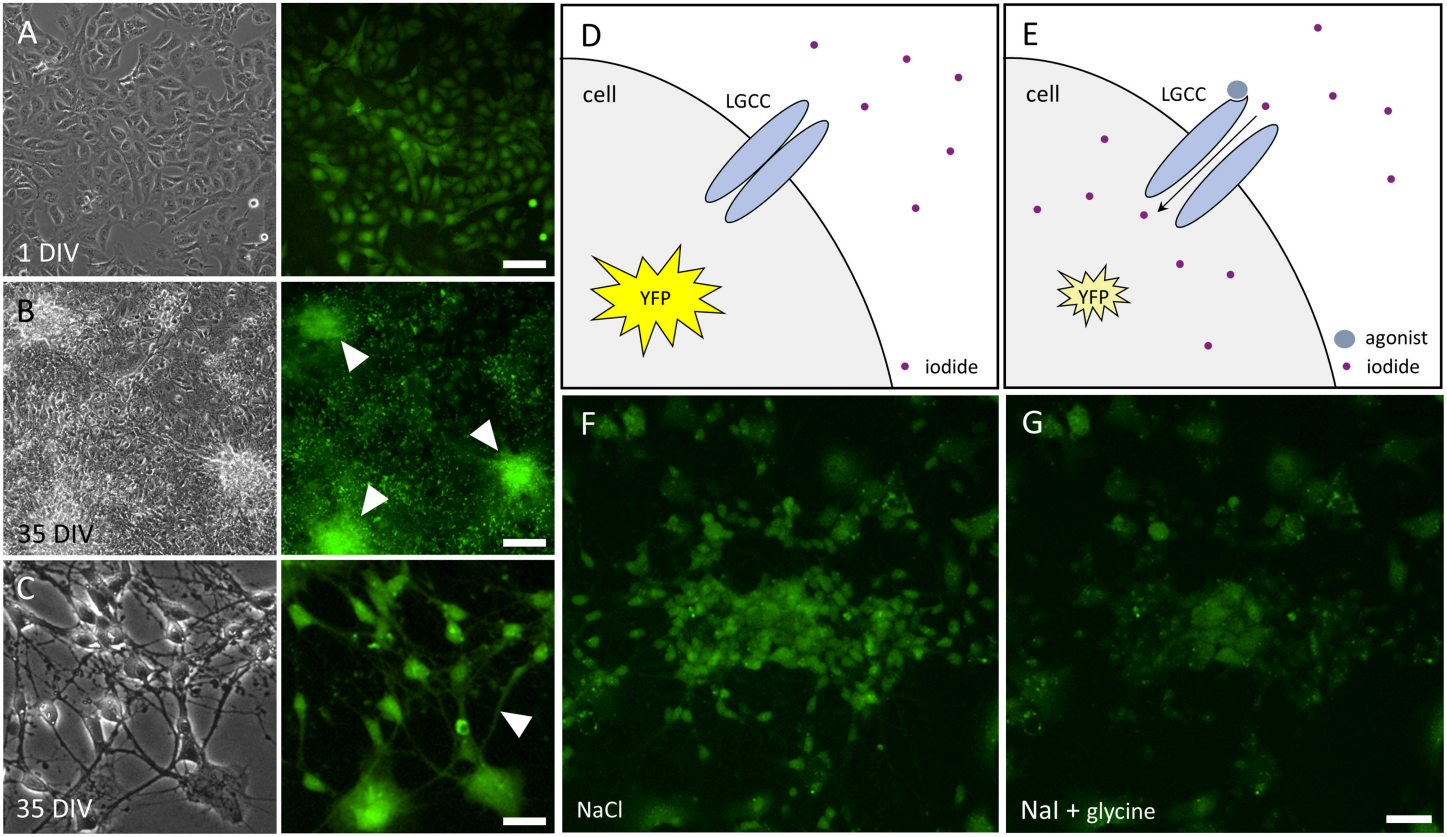
**Images of NT2 and NT2-N cells stably expressing YFP-I152L and principle of functional imaging. (A)** Transmission light (left) and fluorescence image (right) of non-differentiated NT2-YFP-I152L cells after 1 day *in vitro* (DIV) indicating YFP-I152L-expression in most cells of the depicted population. Scale bar 100 μm. **(B,C)**. Transmission light (left) and fluorescence images (right) of differentiated NT2-N-YFP-I152L cells after 35 days *in vitro*. Cell clusters **(B)** and neurites **(C)**, highlighted by white arrows in the respective fluorescence micrographs, indicate successful differentiation of NT2-YFP-I152L cells into NT2-N-YFP-I152L cells and demonstrate that stable integration of the YFP-I152L-gene does not interfere with pluripotency of NT2 stem cells and their ability to undergo neuronal differentiation. Scale bar: 100 μm **(B)** and 30 μm **(C)**. **(D,E)** Principle of fluorescence-based functional imaging of ligand-gated chloride channels (LGCC) such as GABA_A_Rs and GlyRs. **(F)** NT2-N-YFP-I152L cells in control solution (NaCl), captured in unquenched state as reflected in the schematic drawing shown in **(D)**. **(G)** NT2-N-YFP-I152L cells in quenched state, recorded upon addition of test solution (NaI) supplemented with the GlyR agonist glycine and as reflected by **(E)**. Scale bar: 50 μm.

We have established a recombinant human pluripotent stem cell line, stably expressing halide-sensitive YFP-I152L under the control of the human ubiquitin C promoter. The cell line allows fluorescence-based GABA_A_R and GlyR-targeted drug screening and *in vitro* NT/DNT testing at different stages of neuronal maturation. The workflow of cell line generation, stem cell differentiation and functional imaging is shown in Figure 1. Images of recombinant non-differentiated NT2-YFP-I152L stem cells and differentiated NT2-N-YFP-I152L cells as well as the principle of functional imaging using the cells are shown in Figure 2.

### Functional profiling of GABA_A_Rs and GLyRs in recombinant NT2-N-YFP-I152L cells

**Figure 3:**
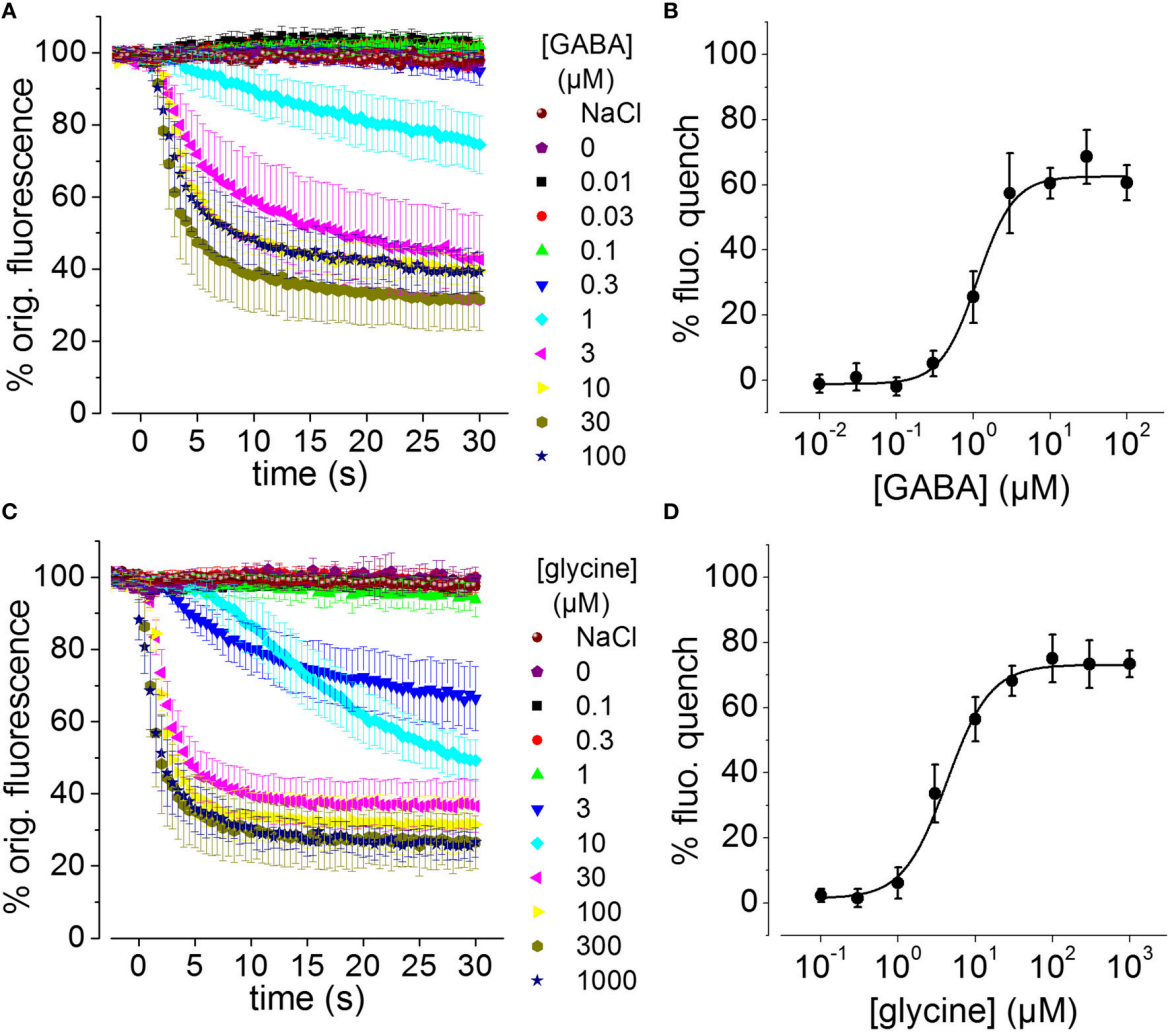
**GABA and glycine-induced fluorescence quench in NT2-N cells stably expressing YFP-I152L measured using a fluorescence microscope. (A,C)** Averaged time-courses of quench following the addition of NaI plus the indicated GABA or glycine concentrations, respectively. **(B,D)** Averaged GABA and glycine concentration–responses measured from the data in **(A,C)**, respectively, 30 s after receptor activation. In this and the subsequent figures, “% fluorescence quench” is defined as (*F*_init_− *F*_final_) × 100 / *F*_init_, where *F*_init_ and *F*_final_ are the initial and final values of fluorescence, respectively. All data are given as mean ± SD (*n* = 10).

To assess whether the generated recombinant stem cell line is suitable to chloride imaging and functional profiling of GABA_A_Rs and GlyRs, NT2-YFP-I152L cells were differentiated into neuronal NT2-N- YFP-I152L cells as described in the Methods section and were seeded at defined density of 2 × 10^4^ cells in each well of a 96-well plate. Two days later and approximately 1 h prior to fluorescence imaging, the culture medium was completely removed and was replaced by 50 μl NaCl control solution. The standard NaCl control solution contained (in mM): NaCl 140, KCl 5, CaCl_2_ 2, MgCl_2_ 1, HEPES 10, glucose 10, pH 7.4 using NaOH. The 96-well plate was placed onto the motorized stage of a high-content imaging system and cells were imaged in control solution to record cellular YFP fluorescence in unquenched state. Because YFP-I152L is almost insensitive to chloride, its fluorescence intensity is highest in NaCl solution allowing for optimal focussing into the optical layer of cells. Subsequently, cells were perfused with 100 μl NaI solution containing increasing concentrations of GABA (0.01–100 μM, Figures 3A,B) or glycine (0.1–1000 μM, Figures 3C,D). The NaI test solution was similar to NaCl control solution except that the NaCl was replaced by equimolar NaI. Cells were imaged throughout the complete procedure of agonist perfusion for a total of 30 seconds and with an acquisition rate of 2 Hz. Fluorescence quench was calculated at single cell level by quantitative analysis as described in the Methods section. The principle of functional profiling of ligand-gated chloride channels is depicted in Figures 2D–G. Average time-courses of quench (mean ± SD, *n* = 10) following the addition of NaI plus the indicated GABA or glycine concentrations are shown in Figures 3A,C, respectively and were constructed by pooling results from wells exposed to different solutions with 10 cells per well. Average agonist dose–response curves constructed from the experiments shown in Figures 3A,C are shown in Figures 3B,D, respectively. Calculated half-maximal activation concentrations (EC_50_) for GABA (1.1 ± 0.2 μM) and glycine (4.3 ± 0.5 μM) are listed in Table 1 and are overall smaller compared with data from functional Cl^−^ imaging and electrophysiology previously reported in the literature (see Discussion). However, Hill coefficients (*n*_H_) for GABA (1.8 ± 0.4) and glycine (1.5 ± 0.2) correspond well with data from the literature. Although EC_50_ values measured in this study are overall smaller compared to values from the literature, these data demonstrate that the cell line is suitable to chloride imaging and functional profiling of GABA_A_Rs and GlyRs.

**Table 1:** **Calculated half-maximal activation concentration and hill coefficients (EC_50_, *n*_H_) for the used neurotransmitters**.

**GABA**				**Glycine**			
**EC_50_ (μM)**	**SD**	***n*_H_**	**SD**	**EC_50_ (μM)**	**SD**	***n*_H_**	**SD**
1.1	0.2	1.8	0.4	4.3	0.5	1.5	0.2

### Toxicological profiling of GABA_A_Rs and GlyRs in recombinant NT2-N-YFP-I152L cells

**Figure 4:**
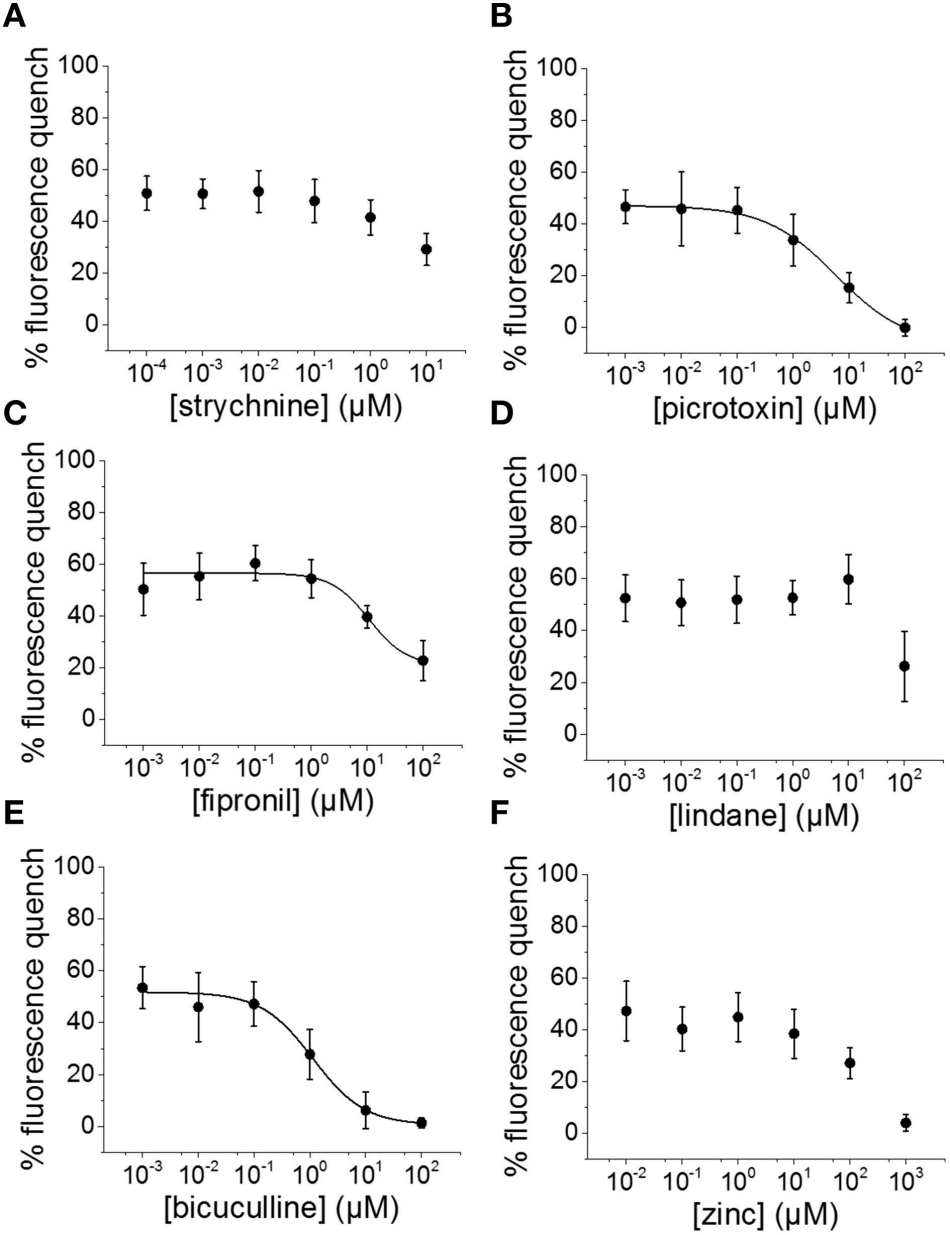
**Applicability of the established *in vitro* model for pharmacological and toxicological evaluation of GABA_A_R modulators. (A–F)** Averaged dose-responses (mean ± SD, *n* = 10) of the chemicals strychnine, picrotoxin, fipronil, lindane, bicuculline, and zinc, demonstrating the applicability of the cell line for GABA_A_R-targeted screening in the context of drug development and NT/DNT testing. 1 μM GABA concentration, basically reflecting GABA half-maximal activation concentration, was used in these experiments.

**Figure 5:**
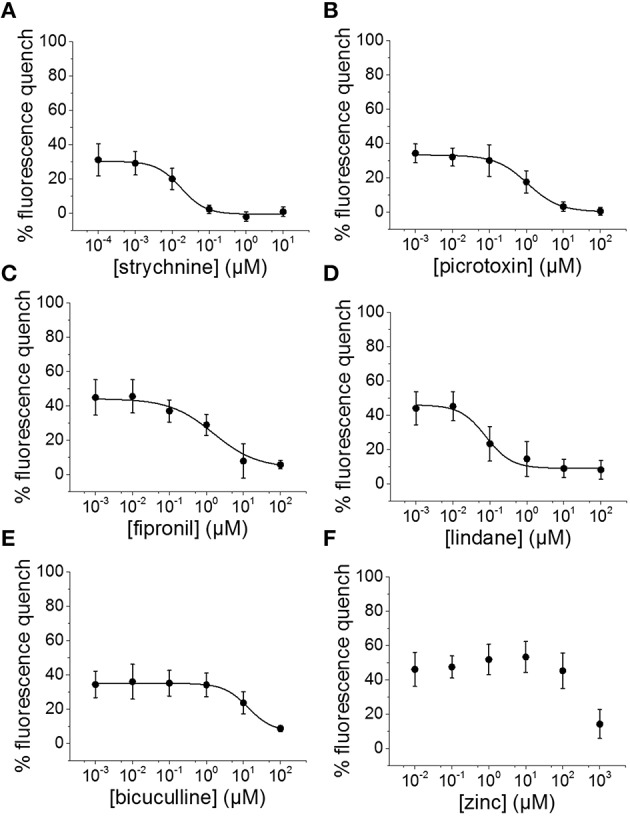
**Suitability of the established cell line for pharmacological and toxicological analysis of GlyR modulators. (A–F)** Averaged dose-responses (mean ± SD, *n* = 10) of the chemicals strychnine, picrotoxin, fipronil, lindane, bicuculline, and zinc. 3 μM glycine concentration, reflecting glycine EC_50_ concentration, was used in these experiments. These data further demonstrate the suitable of the generated *in vitro* model to LGCC-targeted screening in the context of drug development and NT/DNT testing.

To evaluate the suitability of the established cell line for systematic screening for GABA_A_R and GlyR modulators, we conducted concentration-response experiments with the chemicals strychnine, picrotoxin, fipronil, lindane, bicuculline, and zinc in combination with the neurotransmitters GABA or glycine. To this end, human pluripotent NT2-YFP-I152L cells were differentiated into neuronal NT2-N-YFP-I152L cells as described in the Methods section and were seeded at a density of 2 × 10^4^ cells in each well of a 96-well plate. 48 h later, the culture medium was completely removed and was replaced by 50 μl NaCl control solution. The cells were imaged in control solution and during perfusion with 100 μl NaI solution containing 1 μM GABA or 3 μM glycine—basically representing half-maximal activation concentrations—and increasing concentrations of the drugs strychnine (100 pM–10 μM), picrotoxin (1 nM–100 μM), fipronil (1 nM–100 μM), lindane (1 nM–100 μM), bicuculline (1 nM–100 μM) and zinc (10 nM–1 mM). Average (mean ± SD, *n* = 10) drug dose–responses are shown in Figures 4, 5. Calculated half-maximal inhibition concentration (IC_50_) for the tested chemicals are summarized in Table 2 and indicate that the recombinant cell line is suitable to GABA_A_R and GlyR-targeted toxicological profiling and identification of ion channel-specific drugs.

**Table 2:** **Calculated half-maximal inhibition concentration (IC_50_) for the tested chemicals indicating that the recombinant cell line is suitable to GABA_A_R and GlyR-targeted toxicological profiling and identification of ion channel-specific drugs**.

**Drug**	**GABA**		**Glycine**	
	**IC_50_**	**SD**	**IC_50_**	**SD**
Strychnine (nM)	–	–	17.2	7
Picrotoxin (μM)	5.8	0.9	1.1	0.2
Fipronil (μM)	10.8	5.8	1.5	0.9
Lindane (nM)	–	–	78.1	32.6
Bicuculline (μM)	1.1	0.3	13.4	2.5
Zinc	–	–	–	–

### Quantification of the proportion of GABA_A_R and GlyR positive NT2-N-YFP-I152L cells

**Figure 6:**
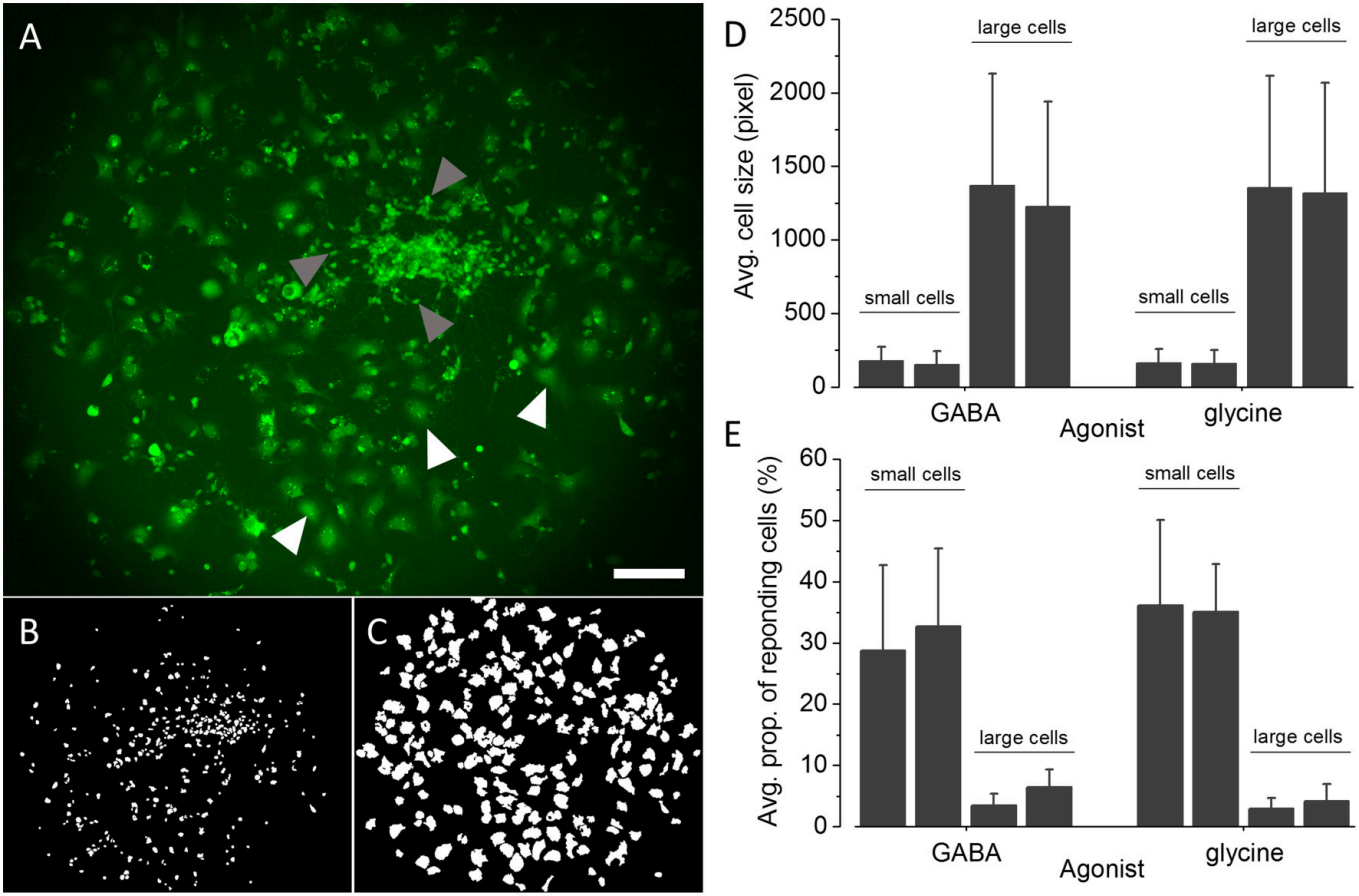
**Quantification of the proportions of GABA_A_R and GlyR-positive NT2-N-YFP-I152L cells. (A)** Representative fluorescence micrograph of NT2-N-YFP-I152L cells in differentiation culture used for functional imaging experiments. **(B,C)** Exemplary segmentation masks created from **(A)** for quantification of “% fluorescence quench” in small NT2-N-YFP-I152L cells (gray arrows, **B**) and large non-neuronal cells (white arrows, **C**), respectively. **(D)** Comparative analysis of the average cell size (in pixel) of GABA_A_R and GlyR-positive small neuronal and large non-neuronal cells quantified from two independent differentiation batches each. **(E)** Comparative analysis of the average percentage of small NT2-N-YFP-I152L and large non-neuronal cells, responding with at least 20% fluorescence quench upon exposure to saturating GABA or glycine concentration, respectively. Approximately one third of the small cell population is represented by GABA_A_R and GlyR-positive cells, equaling about 20% of the overall population of cells. These data indicate a small proportion of GABA_A_R and GlyR-expressing cells that is stable between individual experiments and differentiation batches, further highlighting the applicability of the established *in vitro* model for systematic GABA_A_R and GlyR-targeted high-throughput pharmacological and toxicological screening in the context of drug development and NT/DNT testing. Scale bar: 200 μm.

The applicability of the established recombinant *in vitro* model for high-throughput GABA_A_R and GlyR-targeted screening in the context of lead identification and NT/DNT testing strongly depends on the proportion of GABA_A_R and GlyR positive NT2-N cells in the whole cell population. A very small percentage of GABA_A_R and GlyR expressing NT2-N cells requires a large number of individual experiments including technical and biological replicates to be conducted in order to obtain statistically sound results, potentially compromising the usability of the established cell line for the envisioned application. To obtain a robust estimate of the proportions of GABA_A_R and GlyR positive NT2-N-YFP-I152L cells in the overall cell population, we calculated the percentage of cells per image indicating a fluorescence quench of at least 20% upon application of saturating GABA or glycine concentration. The threshold of 20% fluorescence quench was chosen because, from our experience, any measured cellular fluorescence change within a range of −20 to 20% reflects biological noise typical to the employed assay. As a starting point toward analysing the proportion of GABA_A_R and GlyR-positive cells we segmented the images of a total of 24 individual experiments from two different differentiation batches (GABA_A_R, batch #1: *n* = 7, batch #2: *n* = 6; GlyR, batch #1: *n* = 6, batch #2: *n* = 5) using a modified version of DetecTIFF^©^ software (Gilbert et al., 2009c) employing two individual sets of parameters for the identification of small neuronal NT2-N-YFP-I152L and larger non-neuronal NT2-YFP-I152L cells. Figure 6A shows a representative fluorescence micrograph of large NT2-YFP-I152L and small NT2-N-YFP-I152L cells, highlighted with white and gray arrows, respectively. Exemplary segmentation masks created from the image shown in Figure 6A for quantitative analysis of fluorescence intensity and calculation of “% fluorescence quench” in small neuronal NT2-N-YFP-I152L cells and large non-neuronal cells are depicted in Figures 6B,C. The average cell size (in pixel, mean ± SD) of GABA or glycine-exposed small and large cells calculated from two independent differentiation batches is shown in Figure 6D and is as follows: GABA-exposed small cells: 177 ± 94 (batch #1, *n* = 1869) and 152 ± 89 pixel (batch #2, *n* = 2560), GABA-exposed large cells: 1369 ± 760 (batch #1, *n* = 1216) and 1228 ± 709 pixel (batch #2, *n* = 1371); glycine-exposed small cells: 163 ± 93 (batch #1, *n* = 1631) and 158 ± 92 pixel (batch #2, *n* = 1071); glycine-exposed large cells: 1356 ± 757 (batch #1, *n* = 1212) and 1316 ± 752 pixel (batch #2, *n* = 828). In a next step, we calculated the percentage of cells per image indicating a fluorescence quench of at least 20% upon application of saturating GABA and glycine concentration (see histogram in Figure 6E) that is as follows: GABA-exposed small cells: 28 ± 13 (batch #1, *n* = 7) and 32 ± 12 % (batch #2, *n* = 6), GABA-exposed large cells: 3 ± 1 (batch #1, *n* = 7) and 6 ± 2% (batch #2, *n* = 6); glycine-exposed small cells: 36 ± 14 (batch #1, *n* = 6) and 35 ± 7% (batch #2, *n* = 5); glycine-exposed large cells: 2 ± 1 (batch #1, *n* = 6) and 4 ± 2% (batch #2, *n* = 5). Approximately one third of the small cell population is represented by GABA_A_R and GlyR-positive cells, equaling 17 ± 8 (GABA, batch #1), 21 ± 9 (GABA, batch #2), 20 ± 9 (glycine, batch #1) and 19 ± 4% (glycine, batch #2) and rendering ~20% of the overall population GABA_A_R and GlyR-positive cells. These data indicate a population of GABA_A_R and GlyR-positive cells that is stable between individual experiments and differentiation batches, highlighting the applicability of the established *in vitro* model for systematic GABA_A_R and GlyR-targeted high-throughput pharmacological and toxicological screening in the context of drug development and NT/DNT testing.

## Discussion

For the sake of feasibility, throughput and cost reasons, GABA_A_R and GlyR-targeted screening is typically conducted in non-neuronal recombinant expression systems assessing individual GABA_A_Rs and GlyRs isoforms in isolation. Such expression systems usually also lack fundamental neuronal genetic programs and cell intrinsic regulators of mature neurons *in vivo*. The initial benefit is often compromised by subsequent time-consuming and cost-intensive re-screens for validation and specificity-evaluation of potential GABA_A_R and GlyR-modulators. In the context of neurotoxicity (NT) or developmental neurotoxicity (DNT) testing, GABA_A_Rs and GlyRs can serve as molecular targets and can contribute to deciphering the molecular mechanisms of toxic effects. However, so far there are no suitable *in vitro* models available which comprehensively reflect the complex situation of the central nervous system *in vivo* and NT/DNT testing is still solely based on animal experimentation that is morally questionable, low in throughput, expensive and of rather poor predictive quality. To address these issues, we have established a recombinant human pluripotent stem cell line, stably expressing YFP-I152L that is advantageous for GABA_A_R and GlyR-targeted screening in the context of lead identification and drug development as well as for NT/DNT testing for several reasons. First, the recombinant cell line allows functional profiling of GABA_A_Rs and GlyRs in neuronal environment, providing fundamental neuronal genetic programs and cell intrinsic regulators critical to reflecting the functional properties of mature neurons *in vivo*. Second, the *in vitro* model is of human origin, allowing for GABA_A_R and GlyR-targeted screening relevant to human physiology, including identification of leads for therapeutic intervention and toxicity evaluation. Third, as the cell line used in this study mimics brain maturation to a certain extent, it allows analysis of the physiological properties of GABA_A_R and GlyR during development of the central nervous system. Fourth, the cell model has been applied over a large range of passages—up to passage number 50 until now—without noticeable loss in YFP-I152L fluorescence intensity, further highlighting its applicability to standardized *in vitro* NT/DNT testing. Finally, differentiation of NT2 cells can be conducted in large scale format allowing for systematic high-throughput experimentation.

We exposed the cell line to GABA or glycine and demonstrated that the *in vitro* model is suitable to functional imaging of both GABA_A_Rs and GlyRs. Despite the fact that Hill coefficients compare well with data from the literature, the calculated half-maximal activation concentration for GABA and glycine is overall smaller compared to EC_50_ concentrations previously measured in NT2-N cells using whole cell patch clamp electrophysiology (Neelands et al., 1998; Gao et al., 2004; Gao and Greenfield, 2005; Coyne et al., 2011). Although it is difficult to isolate the reason for this without directly comparing concentration-response data from the same cells generated with different methodologies, the apparent high GABA and glycine sensitivities may have been caused by the relatively long incubation time in agonist solution, i.e., the time between application of the agonist and quantification of the fluorescence intensity (30 s). Furthermore, we have previously observed differences in glycine EC_50_, i.e., increased sensitivity for glycine, in recombinantly expressed α1, α2, and α3 GlyR, assessed with the fluorescence-based assay vs. patch clamp electrophysiology (Talwar et al., 2013).

We used the recombinant *in vitro* model in a case study with the drugs strychnine, picrotoxin, fipronil, lindane, bicuculline and zinc. The imaging data indicate that YFP-I152L-expressing NT2-N cells are applicable to GABA_A_R and GlyR-targeted toxicological profiling and identification of ion channel-specific drugs. The drug strychnine specifically inhibited glycine but not GABA-induced anion-influx with average IC_50_ values that correspond very well with results measured by either electrophysiological or equilibrium [^3^H]-strychnine displacement studies using recombinantly expressed homomeric wildtype α1 GlyRs and variants (Lynch et al., 1997; Vafa et al., 1999). Also, concentration-response experiments with pictrotoxin, a standard pharmacological tool for identifying the presence of β-subunits in recombinant and native GlyRs (Lynch, 2004) revealed half-maximal inhibition concentrations and slope values for glycine and GABA-induced anion influxes, that correspond well with results measured by patch clamp electrophysiology in recombinantly expressed homomeric a2 GlyR (Wang et al., 2007) as well as in human pluripotent stem cell-derived neurons (James et al., 2014), respectively. The alkaloid is known to strongly inhibit GABA_A_Rs at low micromolar concentrations, whereas GlyRs *in vivo* are considered much less sensitive (Lynch, 2004). However, we observed a reversed sensitivity sequence with a picrotoxin-sensitivity of GlyR that is approximately 5-fold higher compared to GABA_A_Rs. As it has been reported that αβ-heteromeric GlyRs were less sensitive to picrotoxin-inhibition than were α-homomeric GlyRs (Pribilla et al., 1992) we speculate that NT2-N-YFP-I152L cells predominantly express α-homomeric GlyR rather than αβ-heteromeric GlyRs, although this is no explanation for the reversed picrotoxin-sensitivities of GlyRs and GABA_A_Rs. Bicuculline, a purported selective antagonist of the GABA_A_ receptor, inhibited the GABA-induced fluorescence quench with a half-maximal inhibition concentration similar to values revealed from rat primary neurons by whole cell electrophysiological recordings (Kumamoto and Murata, 1995). Bicuculline also inhibited glycine induced I^−^ influx in NT2-N-YFP-I152L cells with about 10-fold lower sensitivity compared to GABA-dependent anion influx, highlighting its specificity to GABA_A_R on the one hand, but also confirming previous reports on its inhibitory action assessed in both recombinantly expressed (Sun and Machu, 2000) and native glycine receptors (Bhattarai et al., 2016) on the other hand. The IC_50_ value for glycine-induced fluorescence quench revealed in presence of increasing concentration of the insecticide lindane is about one order of magnitude smaller compared to electrophysiological recordings from recombinantly expressed GlyRs (Islam and Lynch, 2012). These data suggest that NT2-N-YFP-I152L cells predominantly express homomeric GlyRs as lindane has proven to selectively inhibit homomeric but not heteromeric GlyRs (Islam and Lynch, 2012; Talwar et al., 2013). This conclusion is further supported by the above mentioned high picrotoxin-sensitivity of GlyRs. Surprisingly, lindane showed no dose-dependent inhibitory activity on GABA-induced anion influx, although lindane-sensitivity of GABA_A_Rs has been reported by many studies conducted in various culture models and cell types (Narahashi, 1996; Ogata et al., 1988; Vale et al., 2003). A study conducted by Belelli et al. (1999) demonstrated no effect on GABA-evoked currents mediated by anesthetic-insensitive wild-type GABA receptors composed of the (rho 1) subunit (Belelli et al., 1999), suggesting expression of GABA_A_ rho 1 receptor in the examined NT2-N-YFP-I152L cells. Indeed there are a number of studies indicating profound changes in subunit expression and pharmacology during neuronal differentiation—similar to developmental changes in GABA_A_R occurring in neurons of the developing central nervous system—and demonstrating a large variety of neurotransmission phenotypes of non-differentiated NT2 and differentiated NT2-N cell *in vitro* (Neelands et al., 1998, 1999; Guillemain et al., 2000). This question however was not further evaluated in the present study. Fipronil, a broad-spectrum insecticide, inhibited both GABA and glycine induced anion influx in NT2-N cells with sensitivities that correspond very well to results measured by patch clamp electrophysiology in recombinantly expressed GlyRs and GABA_A_R (Ratra et al., 2001; Li and Akk, 2008; Islam and Lynch, 2012). Zinc is widely recognized as a modulator of both, GABAAR and GlyR (Smart et al., 1994). For GlyRs, potentiation as well as inhibition of glycine-activated currents by low (< 10 μM) and high concentrations (>100 μM) of zinc, respectively has been reported (Harvey et al., 1999). For GABAARs, inhibition has been shown at concentrations >10 μM (Kumamoto and Murata, 1995). Although we observed a minor increase of a glycine-induced fluorescence quench (Approximately 10%) in presence of 1 and 10 μM zinc, this effect is not significant. In contrast, zinc-dependent inhibition was observed at concentrations >100 μM for both GABA and glycine-induced anion influx, confirming previous reports on zinc-sensitive GABA-induced currents recorded from NT2-N cells (Gao et al., 2004). The zinc IC_50_ could not be calculated due to a too small range of applied concentrations but presumably exceeds 1 mM, suggesting the presence of γ subunits which have shown to confer zinc insensitivity to αβ GABA_A_Rs (Smart et al., 1991).

We quantified the proportion of GABA_A_R and GlyR positive NT2-N-YFP-I152L cells based on images of the cells and we identified a percentage of approximately 20% of cells expressing GABA_A_Rs and GlyRs, respectively. Despite the fact that the proportion of glycine and GABA sensitive cells are comparable, it cannot be concluded from our data whether the cells are simultaneously sensitive to both glycine and GABA or respond to either of the neurotransmitters. Although the calculated proportions have proven stable between different batches of differentiation cultures, the total percentage of GABA_A_R and GlyR positive NT2-N-YFP-I152L cells is not optimal for plate reader, i.e., photomultiplier or cell population-based screening, that typically requires a high number of reacting, e.g., GlyR or GABA_A_R-positive cells and allows experimentation in high-throughput screening mode. If desired or required, the proportion of NT2-N-YFP-I152L cells may be increased by size-exclusion filtering using e.g., a simple nylon filter of defined mesh size or even more sophisticated cell sorting approaches. As highlighted by gray and white arrows in the fluorescence micrograph depicted in Figure 6A and the histogram shown in Figure 6D, NT2-N cells are much smaller compared to non-neuronal cells, presumably facilitating size-dependent population-increase of NT2-N cells. Despite the fact that plate reader-based screening allows experimentation in high or even ultra-high-screening mode it is disadvantageous with regard to cellular heterogeneity. Cultures of NT2 cells have been reported to express a large variety of non-neuronal and neuronal phenotypes (Guillemain et al., 2000), as we have shown in the case study using drugs with known and partly GABA_A_R and GlyR-specific pharmacological and toxicological profiles. Besides their neuronal properties and human origin, the heterogeneity of NT2-N cell cultures is probably one of the most important advantages over homogeneous, e.g., recombinant expression systems, as it reflects the *in vivo* situation to a much higher extent than simplifying *in vitro* models. Thus, microscopy-based high-content imaging of NT2-N cells, evaluating the physiological pharmacological and toxicological properties at single cell level, and in the context of cellular heterogeneity, is likely to be the optimal approach for systematic GABA_A_R and GlyR-targeted drug screening as well as NT/DNT testing.

To confirm the validity of the morphology or size-based selection criterion, we conducted functional imaging experiments with non-treated NT2-YFP-I152L stem cells and quantified the average cell size as well as the percentage of stem cells, responding with at least 20% fluorescence quench upon exposure to saturating GABA or glycine concentration, respectively (see Supplementary Figure S1). The average cell size (pixel), calculated from two individual experiments each (see Supplementary Figure S1C) is comparable between experiments (GABA, replicate #1: 397 ± 254 pixel, *n* = 650 cells, replicate #2: 385 ± 252 pixel, *n* = 547 cells; glycine, replicate #1: 403 ± 243 pixel, *n* = 589, replicate #2: 400 ± 256 pixel, *n* = 654) and differs from the size of small and large cells, respectively, as indicated in Figure 6D. The percentage of non-treated NT2-YFP-I152L stem cells, responding with at least 20% fluorescence quench upon exposure to 1 mM saturating GABA (replicate #1: 0.62%, replicate #2: 0.37%) or glycine (replicate #1: 0.68%, replicate #2: 0.92%) concentration, respectively (see Supplementary Figure S1D), also differs considerably from data obtained with retinoic acid-exposed NT2-N-YFP-I152L cells. These data clearly demonstrate that the morphology, e.g., the size, as well as the functional properties, i.e., the sensitivity to GABA or glycine, vary between non-treated stem cells and NT2-YFP-I152L differentiation cultures containing small neuronal and large non-neuronal cells and confirming the validity of the employed size-based selection criterion.

We have previously published a method allowing for assessment of ligand-gated ion channels in the context of cellular heterogeneity that is based on progressive receptor activation and iterative fluorescence imaging (Talwar et al., 2013). This method could easily be adapted for application with the established *in vitro* model and has the power to deliver imaging data of unsurpassed functional content presumably outranging any other existing technique for assessing physiological properties of ion channels with regard to throughput.

Due to the fact that our method is based on microscopic evaluation using a single fluorescence indicator, it allows additional fluorescence or luminescence-based markers to be implemented for multiplexing, such as ion or pH-sensitive probes e.g., for parallel analysis of further ion channels or transporters, to assess the activity of intracellular, e.g., toxicity-relevant signaling pathways, or for mapping cellular morphology to functional phenotypes.

It is important to mention that the applicability of the cell line as well as the experimental approach depends on the proteins to be evaluated and targeted, on the individual experimental setup, the available instrumental infrastructure and the biological question to be assessed. While this work focuses on functional profiling and GABA_A_R and GlyR-targeted drug screening as well as neurotoxicity and developmental neurotoxicity, our *in vitro* model and methodological approach could also be adapted for other proteins and strategies, such as RNAi or combined compound and RNAi screening, for single-endpoint or time-resolved functional expression analysis or for approaches using overexpression libraries. Altogether, this work contributes to furthering the applicability of cell-based high-throughput functional screening and provides a means for large-scale characterization of neuronal proteins in the context of *in vitro*-based NT/DNT prediction thus, promoting a systems-level understanding of human physiology in homeostasis and disease.

## Methods

### Pharmacological reagents

Glycine, GABA, strychnine, picrotoxin, fipronil, lindane, zinc chloride and bicuculline were obtained from Sigma. Stock solutions of glycine (1 M), GABA (1 M) and zinc chloride (1 M) were prepared in water, strychnine (10 mM), picrotoxin (100 mM), fipronil (30 mM), lindane (30 mM) and bicuculline (100 mM) were dissolved in dimethylsulphoxide (DMSO). All stocks were frozen at −20°C. From these stocks, solutions for experiments were prepared on the day of recording.

### Reagents for differentiation of NT2 pluripotent stem cells

Retinoic acid and poly-D-lysine (PDL) were obtained from Sigma. Retinoic acid was prepared as 100 mM stock in DMSO. Uridine was prepared as 100 mM stock in water. These stocks were kept at −80 to −20°C. PDL was prepared as 10x stocks in water and stored at 4°C. Solutions for differentiation cultures were prepared freshly at the days of experimentation.

### Molecular constructs

The regulatory region of the human ubiquitin C promoter (1204 bp) was amplified by PCR, the vector pFUGW (Addgene) was used as the template as well as specific primers (Invitrogen). The fragment was then inserted into the promoterless vector pDsRed2-1 (Clontech). PCR of the YFP-I152L gene (720 bp) was performed using vector pcDNA3.1-YFPI152L (Invitrogen) as a template and specific primers (Invitrogen). The YFP-I152L-coding construct was kindly provided from Prof. Joe Lynch (Queensland Brain Institute, The University of Queensland, Brisbane, Australia). After removing the sequence of the red fluorescent protein from the vector phuUbC-DsRed2, YFP-I152L was sub-cloned into the vector. The expression of YFP-I152L is under the control of the constitutive active human ubiquitin C promoter, the plasmid carries the neomycin resistance gene for the selection with the antibiotic geneticin.

### Cell line

Human pluripotent teratocarcinoma NT2 cells (NTERA-2 cl.D1, CRL-1973™) were purchased from *The American Type Culture Collection* (ATCC).

### Generation of stable cell line

For generating a cell line stably expressing YFP-I152L, NT2 stem cells were seeded into 60 mm culture dishes (TPP) at a density of 5 × 10^5^ cells and were incubated at standard conditions over night. The next day, cells were transfected with ubiquitin-YFP-I152L-encoding vector using the calcium phosphate precipitation method. YFP-I152L-positive cells were selected over 5 weeks with 0.5 mg/ml geneticin (Roth, Germany). The geneticin concentration had previously been examined in a concentration-response experiment with 0.1–1.0 mg/ml geneticin by measuring the fluorescence intensity of Hoechst 33342 stained (1 μM) cells exposed to geneticin for 48 h in 96-well plate format using a VICTOR X4 plate reader (Perkin Elmer). The kill curve for evaluation of the geneticin concentration is shown in Figure 1A, subpanel #2. Geneticin-selected NT2-YFP-I152L cells with strongest fluorescence signal were enriched by flow cytometry (FACS, approximately top 30%, see Figure 1A, subpanel #3) in a single step. Enriched cells were maintained in geneticin-supplemented medium at the above mentioned concentration until initiation of the differentiation procedure. Recombinant cells were used over a large range of passages between passage number 15 and 50, without noticeable decline in YFP-I152L fluorescence intensity.

### Cell culture

Cells were maintained in DMEM (Invitrogen) supplemented with 10% fetal bovine serum (FBS, Biochrom) and penicillin (100 U/ml)/streptomycin (100 mg/ml) (Invitrogen) and were cultured in T75 flasks (TPP) at 37°C, 5% CO2 in a humidified incubator according to standard procedures. Cells were passaged every 2–3 days and used in differentiation cultures when approximately 80–90% confluent.

### Differentiation culture

We have previously developed an optimized method for neuronal differentiation of human pluripotent NT2 stem cells in monolayer cultures. In brief, NT2 cells were seeded at a defined density into PDL coated 60 mm dishes (TPP) in standard culture medium supplemented with 10% FBS and penicillin (100 U/ml)/streptomycin (100 mg/ml) and 10 μM RA and were cultured for 5 weeks at 37°C, 5% CO_2_ in a humidified incubator. In the second week, 10 μM uridine was added to the media.

### Preparation of cells for experiments

Following retinoic acid treatment, cells were replated into T25 flasks and cultured overnight at standard conditions. The next day, NT2-N-YFP-I152L cells located on top of non-differentiated cells were removed by tapping and were seeded at a density of 2 × 10^4^ into the wells of a BD Matrigel™ matrix (BD) coated 96-well plate. Cells were used in functional imaging experiments 48 h later.

### Preparation of imaging experiments

Approximately 1 h prior to commencement of experiments culture media in 96-well plates was removed manually and was replaced by 50 μl standard control solution, which contained (in mM) NaCl 140, KCl 5, CaCl_2_ 2, MgCl_2_ 1, HEPES 10, and glucose 10 (pH 7.4, NaOH) upon washing the cells once in 50 μl standard control solution. The NaI test solution was similar in composition to NaCl control solution except the NaCl was replaced by equimolar NaI. For imaging experiments the agonist NaI test solution was supplemented with 1 mM glycine or GABA and was serially diluted with NaI test solution to obtain agonist solutions containing 0.01, 0.03, 0.1, 0.3, 1, 3, 10, 30, 100 μM final GABA or 0.1, 0.3, 1, 3, 10, 30, 100, 300 and 1000 μM final glycine concentration. For antagonist concentration-response experiments, the control solution was supplemented with 1 μM GABA or 3 μM glycine and increasing concentrations of either of the drugs strychnine (100 pM–10 μM), picrotoxin (1 nM–100 μM), fipronil (1 nM–100 μM), lindane (1 nM–100 μM), bicuculline (1 nM–100 μM) and zinc (10 nM–1 mM). All drugs were diluted from stocks at the day of the experiment and experiments were conducted at room temperature.

### Imaging infrastructure

The 96-well plate was placed onto the motorized stage of a high-end long-term imaging system (Nikon Eclipse Ti, Nikon, Japan) and was imaged with a 10x objective (CFI Plan Fluor DL 10X Phase, N.A. 0.30, Nikon, Japan). Illumination from a xenon lamp (Lambda LS, Sutter Instruments, USA), passing through a filter block (C-FL Epi-FL FITC, EX 465-495, DM 505, BA 515-555, Olympus, Japan) was used to excite and detect YFP fluorescence signal. Fluorescence was imaged by a sCMOS camera (NEO, Andor, Ireland) and digitized to disk onto a personal computer (Dell Precision T3500, Dell, USA) with Windows 7 operating System (Microsoft Corporation, USA). The primary resolution of the camera was 2560 × 2160 pixel, although images were binned (2 × 2), resulting in a resolution of 1280 × 1080 pixel. Each image typically contained 350–700 cells. The CCD image acquisition rate was 2 Hz.

### Imaging experiments

The experimental protocol involved imaging each well for 30 s at 2 Hz acquisition rate capturing the initial fluorescence intensity in the control situation as well as the test situation upon receptor activation, respectively. Liquid handling was performed manually.

### Single cell-based quantitative image analysis

Cells depicted in fluorescence micrographs were selected manually based on strongest quench for low and intermediate concentrations and based on cell morphology in experiments where high drug concentrations were applied. Registered images of fluorescent cells were segmented and quantitatively analyzed using NIS-Elements software (Nikon, Japan). The fluorescence signal of identified cells was measured in images acquired before and after the addition of agonist solution as the mean of all pixel values within the area of a cell. “% fluorescence quench” in chloride imaging experiments is defined as

$$\%fluorescence\;quench=\left(F_{init}-F_{final}\right)*100/F_{init}$$

where *F*_*init*_ and *F*_*final*_ are the initial and final values of fluorescence, respectively.

### Calculation of concentration-response relationships

Individual concentration responses were constructed by pooling results from ten cells in one well exposed to agonist solution. Concentration-response relationships were fitted with the following equation:

$$F=\;Fmax+\frac{Fmin-\;Fmax}{1+{\left([Agonist]/EC50\right)}^{nH}}$$

where *F* is the fluorescence corresponding to a particular agonist concentration, [*Agonist*]; *Fmin* and Fmax are the minimal and maximal fluorescence values, respectively; *EC*_50_ is the concentration that elicits half-maximal activation; and *nH* is the Hill coefficient. Curve fits were performed using a least squares fitting routine (Origin 7G, OriginLab Corporation). All averaged results are expressed as mean ± SD.

### Quantification of the proportion of GABA_A_R and GlyR-positive cells

Images of fluorescent cells were segmented and quantitatively analyzed using a modified version of DetecTIFF® software (Gilbert et al., 2009c). In brief, images were segmented using an iterative size and intensity-based thresholding algorithm and the fluorescence signal of identified cells was calculated as the mean of all pixel values within the area of a cell.

### Data analysis and visualization

Imaging data were annotated in Microsoft Excel and analyzed using Origin 7G (OriginLab Corporation).

## Author contributions

KK, OF, and DG conceived the project. KK conducted experiments. KK and DG analyzed the data. KK and DG wrote the paper. All authors commented and agreed on the manuscript.

### Conflict of interest statement

The authors declare that the research was conducted in the absence of any commercial or financial relationships that could be construed as a potential conflict of interest.
