# Supplementary material for: A Recombinant Human Pluripotent Stem Cell Line Stably Expressing Halide-Sensitive YFP-I152L for GABAAR and GlyR-Targeted High-Throughput Drug Screening and Toxicity Testing
Source: Front Mol Neurosci. 2016 Jun 28;9:51. doi: 10.3389/fnmol.2016.00051 (PMC4923258; doi:10.3389/fnmol.2016.00051)
Supplement: Supplementary file 1 [file DataSheet1.PDF]

A recombinant human pluripotent stem cell line stably expressing halide-sensitive YFP-I152L for $GABA_AR$ and GlyR-targeted high-throughput drug screening and toxicity testing

Katharina Kuenzel[a,b], Oliver Friedrich[a,b] and Daniel F. Gilbert*[,a,b]

[a] *Institute of Medical Biotechnology, Friedrich-Alexander-Universität Erlangen-Nürnberg, Erlangen, Germany.*
[b] *Erlangen Graduate School in Advanced Optical Technologies (SAOT), Friedrich-Alexander-Universität Erlangen-Nürnberg Erlangen, Germany.*
** Correspondence to: Dr. Daniel Gilbert, daniel.gilbert@mbt.uni-erlangen.de*

Supplementary Information – Figure S1

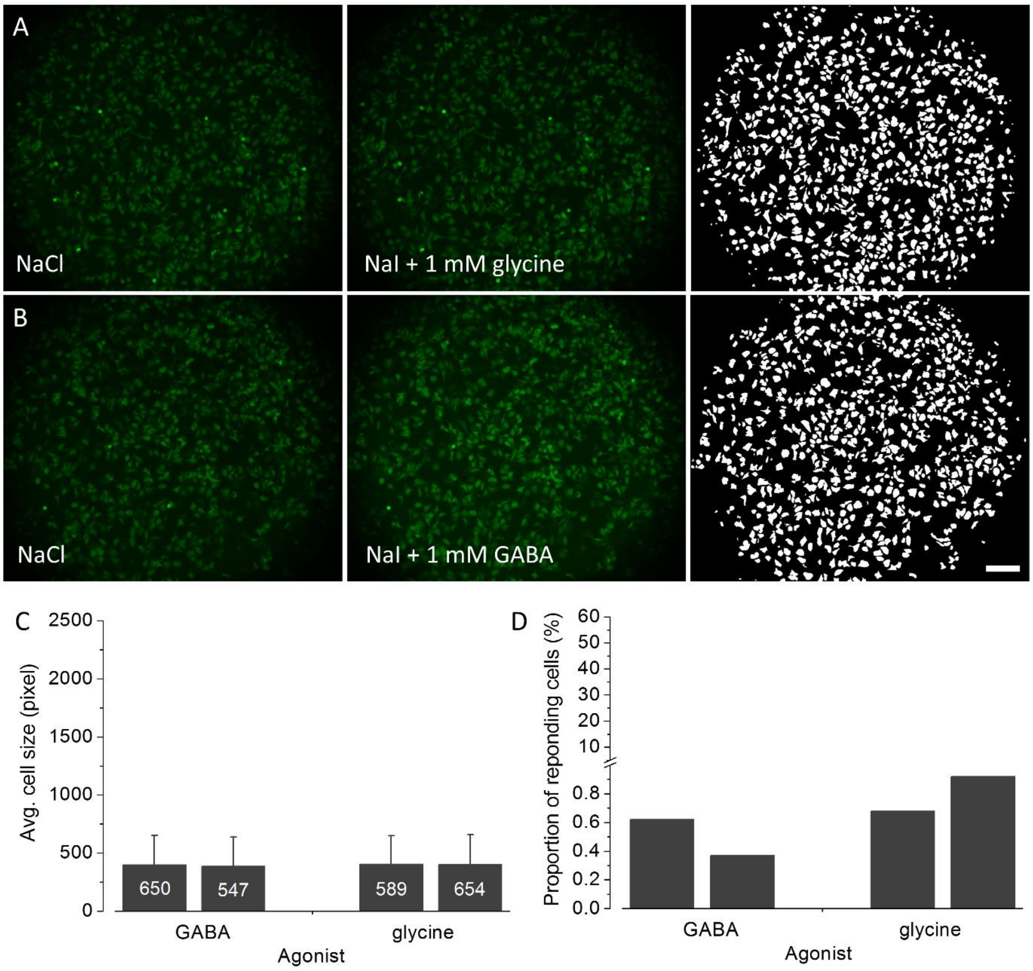

Figure S1. Functional imaging with non-treated NT2-YFP-I152L stem cells. A and B. Representative fluorescence micrographs taken from non-treated NT2-YFP-I152L stem cells under control conditions (left) and 30s after application of NaI solution supplemented with 1 mM glycine (A, middle) or 1 mM GABA (B, middle) and associated binary masks (right) derived from image analysis software for quantitative analysis. **C.** Averaged cell size (pixel) calculated from two individual experiments each, conduced with both agonists. The white value indicated in the histogram bars represents the number of cells identified in fluorescence micrographs. These data demonstrate that the average cell size is

comparable between experiments and differs from the size of small and large cells, respectively, as indicated in Fig. 6D. **D.** Analysis of the percentage of non-treated NT2-YFP-I152L stem cells, responding with at least 20% fluorescence quench upon exposure to 1 mM saturating GABA or glycine concentration, respectively. The calculated percentages of non-treated NT2-YFP-I152L stem cells exposed to 1 mM saturating GABA or glycine concentration, respectively, also differs considerably from data obtained with retinoic acid-exposed NT2-N-YFP-I152L cells. These data further demonstrate that the morphology (size) as well as the functional properties (sensitivity to GABA or glycine) differ from NT2-YFP-I152L differentiation cultures containing small neuronal and large non-neuronal cells, validating the employed size-based selection criterion for discrimination of neuronal and neuronal cells in NT2-YFP-I152L differentiation cultures. Scale bar: 200 µm.
